# Effect of Galacto-Oligosaccharides: Maltodextrin Matrices on the Recovery of *Lactobacillus plantarum* after Spray-Drying

**DOI:** 10.3389/fmicb.2016.00584

**Published:** 2016-05-03

**Authors:** Natalia Sosa, Esteban Gerbino, Marina A. Golowczyc, Carolina Schebor, Andrea Gómez-Zavaglia, E. Elizabeth Tymczyszyn

**Affiliations:** ^1^Facultad de Bromatología, Universidad Nacional de Entre RíosGualeguaychú, Argentina; ^2^Centro de Investigación y Desarrollo en Criotecnología de Alimentos (CIDCA, CCT-CONICET)La Plata, Argentina; ^3^Departamento de Industrias, Facultad de Ciencias Exactas y Naturales, Universidad de Buenos Aires, Ciudad UniversitariaBuenos Aires, Argentina; ^4^Laboratorio de Microbiología Molecular, Departamento de Ciencia y Tecnología, Universidad Nacional de QuilmesBernal, Argentina

**Keywords:** galacto-oligosaccharides, maltodextrin, glass transition temperature, spray-drying, lactic acid bacteria

## Abstract

In this work maltodextrins were added to commercial galacto-oligosaccharides (GOS) in a 1:1 ratio and their thermophysical characteristics were analyzed. GOS:MD solutions were then used as matrices during spray-drying of *Lactobacillus plantarum* CIDCA 83114. The obtained powders were equilibrated at different relative humidities (RH) and stored at 5 and 20°C for 12 weeks, or at 30°C for 6 weeks. The T_g_s of GOS:MD matrices were about 20–30°C higher than those of GOS at RH within 11 and 52%. A linear relation between the spin-spin relaxation time (T_2_) and T-T_g_ parameter was observed for GOS:MD matrices equilibrated at 11, 22, 33, and 44% RH at 5, 20, and 30°C. Spray-drying of *L. plantarum* CIDCA 83114 in GOS:MD matrices allowed the recovery of 93% microorganisms. In contrast, only 64% microorganisms were recovered when no GOS were included in the dehydration medium. Survival of *L. plantarum* CIDCA 83114 during storage showed the best performance for bacteria stored at 5°C. In a further step, the slopes of the linear regressions provided information about the rate of microbial inactivation for each storage condition (*k* values). This information can be useful to calculate the shelf-life of spray-dried starters stored at different temperatures and RH. Using GOS:MD matrices as a dehydration medium enhanced the recovery of *L. plantarum* CIDCA 83114 after spray-drying. This strategy allowed for the first time the spray-drying stabilization of a potentially probiotic strain in the presence of GOS.

## Introduction

Galacto-oligosaccharides (GOS) are non-digestible oligosaccharides composed of a variable number of galactose units (usually from 2 to 10) and a terminal glucose unit, linked mostly by β1-4 and β1-6 bonds (Casci and Rastall, 2006; Vera et al., 2011). They are widely known because of their prebiotic properties^1^ (Gibson and Roberfroid, 1995) and more recently, they demonstrated to enhance the recovery of lactic acid bacteria during dehydration processes (Tymczyszyn et al., 2011, 2012; Santos et al., 2014a,b,c). The protective properties of GOS have been explained on the basis of their ability to form glassy matrices of high viscosity and low molecular mobility where molecular interactions are restricted (Tymczyszyn et al., 2011, 2012; Golowczyc et al., 2013; Santos et al., 2014a,b,c).

Commercial GOS are generally produced by transgalactosylation of lactose in reactions catalyzed by fungal and bacterial β-galactosidases (Gänzle et al., 2008). As a result of transgalactosylation, GOS of different degrees of polymerization are the main products of reaction, and glucose and lactose remain as secondary products (about 20–25% of the obtained products) (Tymczyszyn et al., 2011). The presence of these latter compounds in the reaction medium leads to a decrease of the glass transition temperatures (T_g_) of the obtained GOS. Even when commercial GOS are efficient lyoprotectants of lactic acid bacteria during freeze-drying, they fail at stabilizing them during storage because of their relatively low T_g_ (Tymczyszyn et al., 2012). Therefore, removing glucose and lactose can contribute to increase the T_g_ of commercial GOS. Different technologies, including chromatographic operations, selective fermentation, or nanofiltration, have been proposed as purification methods (Cheng et al., 2006; Goulas et al., 2007; Li et al., 2008; Feng et al., 2009; Sanz-Valero, 2009; Botelho-Cunha et al., 2010; Gosling et al., 2010). However, they are expensive for a large scale production, and in practice, are impracticable for small and medium enterprises. Considering the economical importance of GOS, other strategies should be developed to increase their T_g_ and thus, improve their capacity to stabilize microorganisms during storage.

Spray-drying is a cost-effective technique that leads to the transformation of liquid systems (*i.e*., solutions, dispersions, emulsions) into dry particulate powders when they get in contact with a drying medium (air) at high temperatures. This technique has been successfully scaled-up, leading to the production of flowable powders and reducing the storage and transportation costs (Santivarangkna et al., 2008). It is commonly used to ensure the stability of food products and has been increasingly used to stabilize lactic acid bacteria (Golowczyc et al., 2010, 2011a,b). Using carbohydrates in the dehydration medium contributes to increase the stability of the spray-dried products and bacteria in terms of water activity, moisture content, pH, solubility, hygroscopicity, nutritional composition, glass transition temperature, color, and fluidity (Chen and Patel, 2008; Fazaeli et al., 2012). However, the thermophysical properties of some commercial GOS are not the most appropriate ones to support their use as dehydration media. Their low T_g_ (Tymczyszyn et al., 2012) leads to obtaining sticky products that agglomerate and adhere to the internal wall of the drying chamber, thus resulting in a low product yield and low bacterial cultivability (Hennigs et al., 2001; Bhandari and Howes, 2005). To overcome this problem, high molecular weight saccharides of high T_g_, including maltodextrins (MD) or starch, have been generally used to avoid the undesirable stickiness of low T_g_ sugar solutions (Bhandari et al., 1997; Fazaeli et al., 2012; Rajam and Anandharamakrishnan, 2015).

Taking into account that commercial GOS generally include different amounts of glucose and lactose, and their purification is a costly process, the addition of MD to the GOS solutions used as dehydration medium appears as an adequate strategy to increase the T_g_ of the powders obtained after spray-drying. For this reason, the goal of this work was to use commercial GOS in combination with MD to stabilize *Lactobacillus plantarum* CIDCA 83114 during spray-drying and storage. The thermophysical properties of GOS:MD matrices were analyzed by determining water sorption isotherms at room temperature, T_g_s by differential scanning calorimetry (DSC), and molecular mobility by proton nuclear magnetic resonance (^1^H-NMR). The investigated matrices were then used as dehydration media during spray-drying of *L. plantarum* CIDCA 83114, a potentially probiotic strain (Hugo et al., 2008; Golowczyc et al., 2011a; Kakisu et al., 2013a,b). The obtained powders were equilibrated at different relative humidities (RH) and stored at different temperatures. The use of MD to increase the Tg of the dehydration matrices allowed for the first time, the spray-drying of a potentially probiotic strain in the presence of GOS.

## Materials and methods

### Galacto-oligosaccharides and maltodextrin

A commercial syrup Cup Oligo H-70® (Kowa Company, Tokyo, Japan) kindly donated by Kochi S.A. (Santiago, Chile) was used in this study. It contains 75% GOS (d.b.) of different degrees of polymerization (DP): 4% high-molecular-weight oligosaccharides (DP ≥ 5); 21% tetrasaccharides (DP4); 47% trisaccharides (DP 3); 23% disaccharides (DP2) including lactose; and 5% monosacharides, including glucose and galactose (Tymczyszyn et al., 2011). Food grade maltodextrin (MD) (dextrose equivalent: 12) was used (Maltodextrin DE 12, Ingredion, Buenos Aires, Argentina).

### Microorganisms

*L. plantarum* CIDCA 83114 was maintained frozen at −80°C in 120 g/L non-fat milk solids. Microbial cells were reactivated in MRS broth (de Man et al., 1960) at 37°C before conducting the experiments. For dehydration experiments, reactivated microorganisms were grown in MRS at 37°C for 24 h (early stationary phase). Then, microorganisms were harvested by centrifugation at 7000 × g at 4°C for 10 min, washed twice with 0.85% w/v NaCl (Merck Química, Buenos Aires, Argentina) and suspended in the same volume of a solution containing 20% w/w GOS and 20% w/w MD.

### Spray-drying procedure

GOS:MD solutions containing microorganisms were spray-dried in a laboratory-scale spray-dryer (model B290 Büchi mini spray-dryer) at a constant air inlet temperature of 180°C and an outlet temperature of 75–80°C. Atomization was created by compressed air at a pressure of 0.5–2 bar and an air flux of 600 L/h. Nozzle diameter was 0.7 mm. Controls: microorganisms harvested in the stationary phase, washed with 0.85% w/v NaCl (Merck Química, Buenos Aires, Argentina), suspended in 40% MD solutions and spray-dried in the same conditions as the samples.

### Humidification procedure

Opened glass vials containing approximately 1 g of spray-dried samples were equilibrated in sealed jars for 15 days at 20°C in atmospheres of the following saturated salts: LiCl, KCH_3_COO, MgCl_2_, K_2_CO_3_, and Mg(NO_3_)_2_ (Sigma-Aldrich, Buenos Aires, Argentina), giving RHs of 11, 22, 33, 44, and 52%, respectively. After having attained the equilibrium, the vials were hermetically closed to be used in the activities explained below.

### Glass transition temperatures (T_g_)

Glass transition temperatures of the spray-dried samples were determined by DSC (onset values, heating rate: 10°C/min) using a DSC 822 Mettler Toledo calorimeter (Schwerzenbach, Switzerland), calibrated with indium, lead and zinc. Hermetically sealed 40 μL medium pressure pans were used (an empty pan was used as reference). Heating and cooling cycles were carried out within the −80 to 90°C range at 10°C /min. Thermograms were evaluated using Mettler Star^e^ program. An average value of at least two replicates was reported. The standard deviation for the glass transition temperature measurement was ±1°C.

### Water content determination

Karl Fischer titration was carried out at 25 ± 1°C with a Karl Fischer titrator DL 31 from Mettler-Toledo (Zurich, Switzerland), applying the one-component technique with Hydranal Titrant Composite 5 from Riedel-de Haën (Seelze, Germany). A 95 (1:1) methanol:formamide mixture, obtained from Merck (Darmstadt, Germany), was used as solvent. Sample sizes were approximately 100 mg.

### Molecular mobility

A Bruker mq20 Minispec pulsed ^1^H-NMR instrument (Bruker Biospin GmbH, Rheinstetten, Germany), with a 0.47 T magnetic field operating at resonance frequency of 20 MHz, was used for measurements. Equilibrated samples were removed from the desiccators, placed into 10 mm diameter glass tubes and returned to the desiccators for 24 h prior to analysis.

The spin-spin relaxation time (T_2_) associated to the fast relaxing protons (related to the solid matrix and to water tightly interacting with solids) was measured using a free induction decay analysis (FID) after a single 90° pulse. The decay envelopes [protons signal intensity (I) vs. experimental time (t)] were fitted to mono-exponential behavior using Equation 1:

(1) $$\begin{array}{l} \text{I }=\;\text{A}\;\text{exp}\left(-{\text{t/T}}_{2}\right) \end{array}$$

where T_2_ is the relaxation time of protons in the polymeric chains of the sample and of tightly bound water, and A is a constant. Since no 180° refocus pulse was used in the experiments, the spin-spin relaxation time constants are apparent relaxation time constants, i.e., ${\text{T}}_{2}^{*}$. For solid samples, like those used in this work, it can be assumed that intrinsic T_2_ is very close to ${\text{T}}_{2}^{*}$ (Fullerton and Cameron, 1988). Therefore, T_2_ was used for convenience.

### Bacterial plate counts

Bacterial cultivability was determined before and after spray-drying and during storage of the equilibrated samples at different temperatures (see below). One gram of spray-dried powder was rehydrated in 9 mL of 0.85% w/v NaCl, homogenized for 1 min in a vortex mixer and maintained at room temperature for 30 min. Bacterial suspensions were serially diluted and plated on MRS agar plates. Bacterial counts were determined after 48 h incubation at 37°C and referred to bacterial counts immediately after spray-drying (N_0_).

Bacterial inactivation rate was determined according to Equation 2:

(2) $$\begin{array}{l} {\text{Log N/N}}_{0}\;=\;-k\text{t} \end{array}$$

where *N* is the CFU/g powder at a given time of storage, *N*_0_ is the CFU/g powder immediately after spray drying, *t* is the time of storage expressed in days, and *k* is the bacterial inactivation constant expressed in days^−1^.

### Storage

Equilibrated samples at 11, 22, 33, and 44% RH were sealed and stored at 5 and 20°C for 12 weeks, or at 30°C for 6 weeks. The recovery of cells after different times of storage was analyzed by plate counts.

The bacterial inactivation constant obtained from Equation (2) (*k*) was then correlated with the inverse of the storage temperature (1/T) and fitted to the Arrhenius equation according to Equation 3:

(3) $$\begin{array}{l} \text{k}\;=\;\text{A}\;e^{-Ea\;∕\;RT} \end{array}$$

The linearization of Equation (3) leads to Equation (4):

(4) $$\begin{array}{l} \text{Ln}\;\text{k }=\;-\;\frac{\text{Ea}}{\text{RT}}+\ln\text{ A } \end{array}$$

where *k* is the bacterial inactivation rate constant, A is a frequency factor in units of time^−1^, Ea is the apparent energy of activation in kJ/mol, R is the gas constant (8.314 J/K mol), and T is the storage temperature in K (Muller et al., 2013).

### Statistical analysis

All experiments were carried out on duplicate samples using three independent cultures of bacteria. Modeling of Arrhenius equation was carried out by two steps linear least squares fit (Cohen and Saguy, 1985) and the linear regressions and the goodness of fit (*R*^2^, residuals and *t*-test) were carried out using GraphPad Prism 5 software (GraphPad Software Inc., San Diego, CA, 2007). The regression was considered statistically significant if *P* < 0.1.

## Results

**Figure 1:**
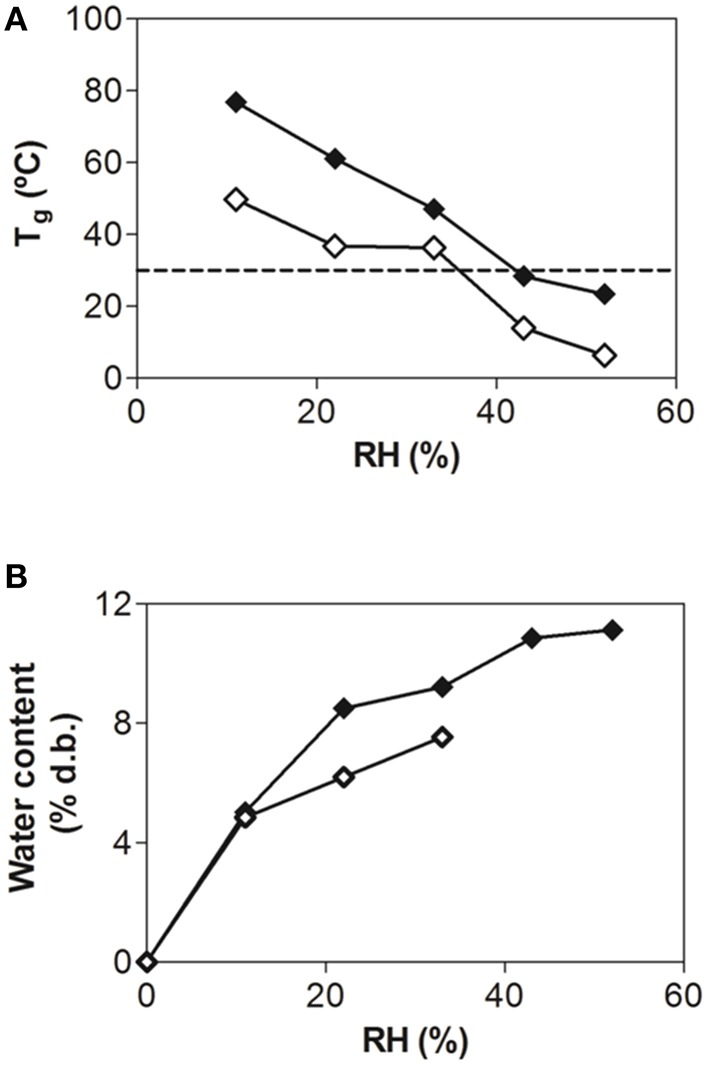
**Onset glass transition temperatures (T_g_) (A) and water content (in d.b.) (B) of the spray-dried GOS:MD (♦) and freeze-dried GOS (♢) matrices (without microorganisms) after equilibration at different RHs (30°C)**. Data corresponding to freeze-dried GOS (♢) were obtained previously in the same conditions (Tymczyszyn et al., 2012), and included in the plot for comparison. The dashed line in **(A)** indicates the maximum storage temperature, 30°C.

Figure 1A depicts the T_g_ of GOS:MD matrices spray-dried in the absence of bacteria and equilibrated at RHs ranging from 11 to 52%. For comparison purposes, information previously obtained for GOS dried without MD was added in the plot (Tymczyszyn et al., 2012). The addition of MD led to a dramatic increase of the T_g_ of GOS at all the RHs assayed (Figure 1A). In this regard, at 11% RH the T_g_ values of GOS:MD were 30°C higher than those of GOS, and at 52% RH an increase of 20°C with regard to GOS was observed (Figure 1A). Figure 1B depicts the water content of equilibrated spray-dried GOS-MD matrices as a function of RH. Previously obtained results for dehydrated GOS in the absence of MD were also displayed for comparison. It is interesting to note that for RHs greater than 11%, the water content of GOS:MD matrices is noticeably higher than that of GOS matrix. It is also important to point out that the addition of MD to commercial GOS allowed the retention of the glassy state up to 44% RH at 30°C (Figure 1A). In contrast, when no MD was added, GOS remained in a glassy state only up to 33% RH at 30°C (Tymczyszyn et al., 2012). These results support the use of GOS:MD formulations as dehydration matrices during bacteria spray-drying.

**Figure 2:**
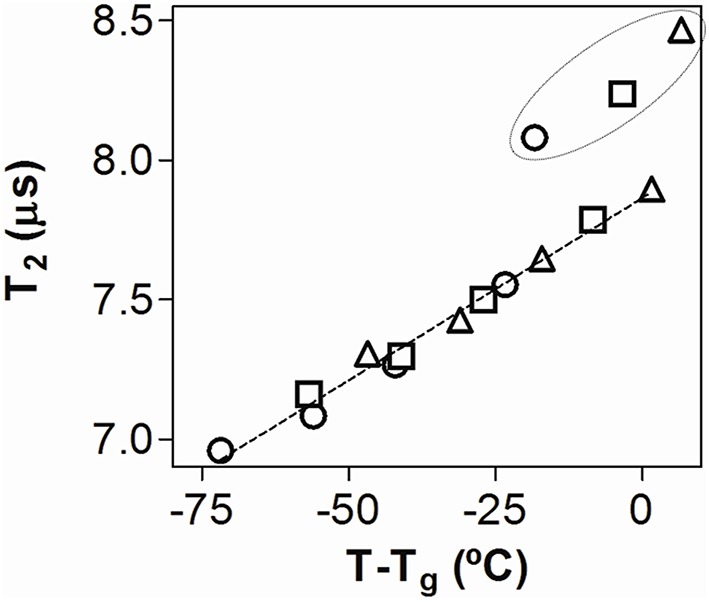
**T_2_ for spray-dried GOS:MD matrices as a function of T-T_g_**. T_g_ were obtained from Figure 1 and T corresponds to the three different storage temperatures: 5°C (○); 20°C (□); and 30°C (△). The points within the ellipse indicate samples equilibrated at 52% RH.

T_2_ showed a linear relation with the T-T_g_ parameter for GOS:MD matrices equilibrated at 11, 22, 33, and 44% RH, at 5, 20, and 30°C (Figure 2). It is interesting to note that samples equilibrated at 52% RH are out of the linear regression (see ellipse in Figure 2). In fact, for these samples a noticeable increase of T_2_ was observed at all the temperatures assayed (5, 20, and 30°C). The rubbery state of all the samples at 52% RH could explain this behavior.

The investigated GOS:MD matrices were then used as dehydration media during spray-drying of *L. plantarum* CIDCA 83114, leading to 93% of bacterial recovery after the thermal treatment (Table 1). A much lower recovery (64%) was observed when only MD was included in the dehydration medium.

**Table 1:** **Recovery of *L. plantarum* CIDCA 83114 after spray-drying in GOS:MD and MD solutions**.

**Matrix**	**Before spray-drying (CFU/g)**	**After spray-drying (CFU/g)**	**Bacterial recovery %**
GOS-MD	1.2 10^11^ ± 0.5 10^10^	1.12 10^11^ ± 3.5 10^9^	93
MD (control)	1.2 10^11^ ± 0.5 10^10^	7.75 10^10^ ± 3.5 10^9^	64

**Figure 3:**
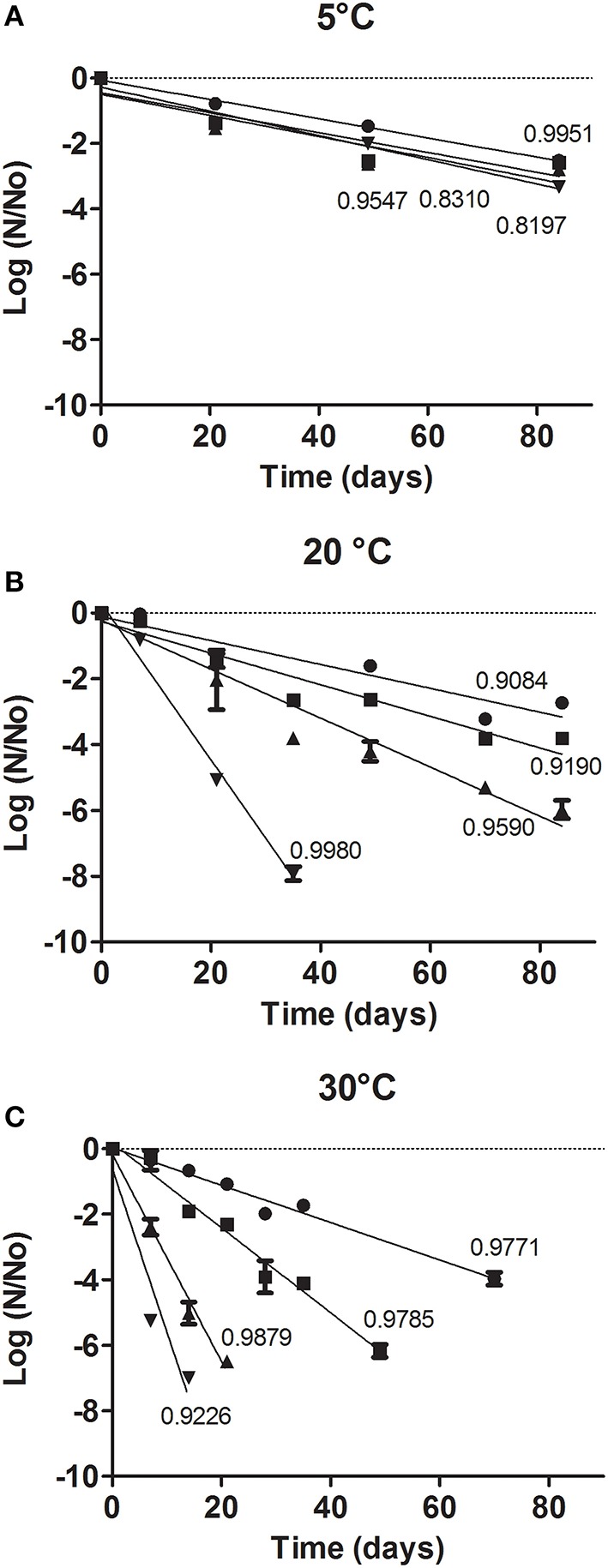
**Relative survival fraction (log N/N_0_) of *L. plantarum* spray-dried in a GOS:MD matrix and stored at: 5°C (A); 20°C (B); 30°C (C)**. For each storage temperature, samples were equilibrated at 11 (●), 22 (■), 33 (▲) and 44 (▼)% RH. N = CFU of humidified samples after storage; N_0_ = CFU after spray-drying. Solid line indicates the linear regression for each condition. The numbers near the lines indicate the *R*^2^ values.

Figure 3 shows the evolution of Log N/N_0_ for *L. plantarum* CIDCA 83114 spray-dried and equilibrated at 11, 22, 33, and 44 % RH, and stored at 5, 20, and 30°C for 12 weeks (N_0_ = 1.12 10^11^ ± 3.5 10^9^ CFU/g). As expected, microorganisms showed the best performance when stored at 5°C (Figure 3A). In turn, 11% RH was the best condition when stored at 20 and 30°C (Figures 3B,C). Storage at 30°C and 44% RH was the most detrimental condition (Figure 3C).

**Figure 4:**
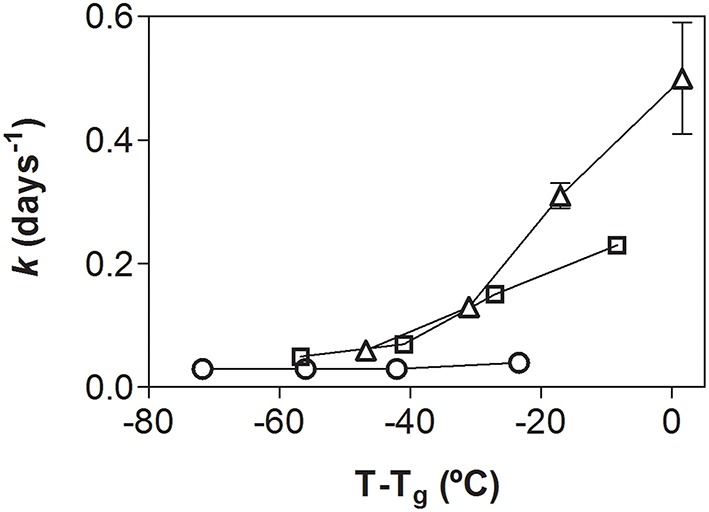
**Rate constant of microbial inactivation (*k*) as a function of T-T_g_**. *k* was obtained from the slopes of the linear regressions plotted in Figure 3. Microorganisms stored at 5°C (○); 20°C (□) and 30°C (△) were included in the plot.

In a further step, the slopes of the linear regressions provided information about the rate of microbial inactivation for each storage condition (*k* values) were correlated with T-T_g_ parameter (Figure 4). A non-linear increase of *k* values was observed as function of T-T_g_ parameter. Although most of the samples were in the glassy state, the inactivation constants showed relatively high values, particularly for the samples stored at the highest temperatures. The *k* values increase was especially evident for samples stored at 30°C (triangles in Figure 4). This result indicates that the storage temperature was more relevant than T_g_ on the inactivation constants.

**Figure 5:**
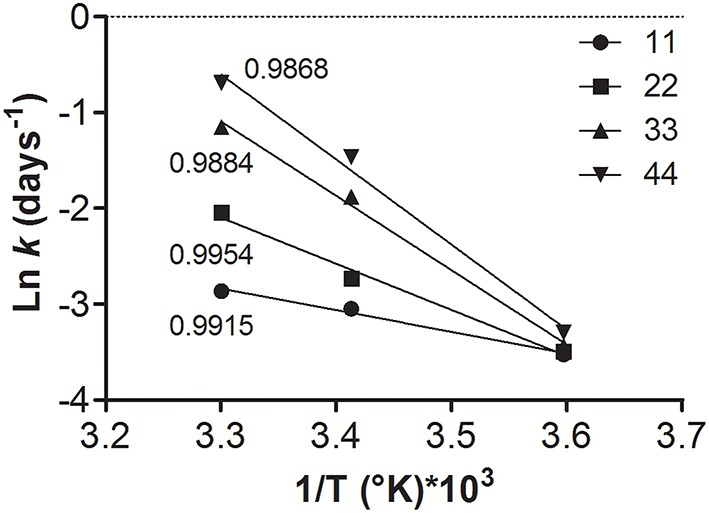
**Arrhenius plot Ln *k* vs. 1/T for each storage condition**. Temperatures are expressed in K. 11% (●); 22% (■); 33% (▲); 44% (▼) RH. Solid lines indicate the linear regressions for each condition. The numbers close to the lines indicate the *R*^2^ values. All fittings were statistically significant (*P* < 0.1).

Figure 5 shows a linear correlation between *k* (obtained from the slopes of Figure 3) and the absolute temperature of storage for each RH. Fitting the Arrhenius equation (Equation 3) allowed determining the bacterial inactivation constants (*k*). In turn, with this parameter it is possible to predict the decrease of viability (log N/N_0_) in a given sample at a given water activity, time, and temperature of storage (Equation 2). The linearization of this plot (4) showed that higher slopes were observed with the increase in RH (Figure 5). This indicates that the higher the storage temperature, the higher their sensitivity to RH.

**Figure 6:**
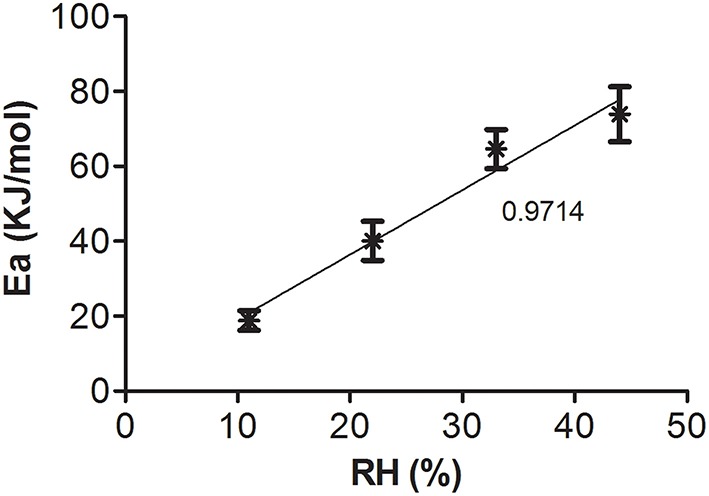
**Activation energy (Ea) as a function of RH**. The number near the line indicates *R*^2^.

From the slopes of the linear regressions obtained in Figure 5, it was possible to calculate the Ea for each storage condition. These Ea also showed a linear relation with RH (Figure 6), and this information can be useful to calculate the shelf-life of spray-dried samples stored at different temperatures and RH (Muller et al., 2013).

## Discussion

The T_g_s of the commercial GOS used in this work were relatively close to the storage temperatures at all the RH essayed (Figure 1A). The addition of MD to the GOS matrices in a 1:1 ratio appeared as an adequate strategy to increase the T_g_ of commercial GOS, leading to the stabilization of *L. plantarum* CIDCA 83114 during spray-drying (Table 1). Very recently, Rajam and Anandharamakrishnan (2015) adopted a similar strategy to stabilize a strain of *L. plantarum* during spray-drying. In the mentioned article, the addition of whey proteins and hydrolyzed whey proteins to fructo-oligosaccharides matrices led to a decrease of stickiness and moisture content of the dehydrated samples (Rajam and Anandharamakrishnan, 2015).

MD are high molecular weight polysaccharides, produced by starch hydrolysis. They have different molecular sizes and are classified as a function of their dextrose equivalent units. Because of their low viscosity, high solid content, adequate water solubility, and high T_g_, they are, in principle, good matrices to stabilize samples during spray-drying (Hogan et al., 2001; Sosa et al., 2011). However, using MD matrices without GOS resulted in a noticeable decrease of bacterial cultivability (Table 1). This indicates that the GOS:MD matrices had a double effect. From one side, MD led to an increase of T_g_s (Figure 1A) and from the other side, the presence of GOS in the matrices led to *L. plantarum* CIDCA 83114 stabilization (Table 1 and Figure 3). Although other sugars, including trehalose, glucose, and inulin, have been previously used to stabilize lactobacilli strains during spray-drying (Sunny-Roberts and Knorr, 2009; Golowczyc et al., 2011b; Ying et al., 2012), up to our knowledge, the thermoprotectant effect of GOS has been reported for the first time in this work.

It is interesting to note that although the addition of MD led to a noticeable increase of T_g_ (Figure 1A), the molecular mobility of GOS:MD was slightly higher than that reported for dehydrated GOS (Tymczyszyn et al., 2012). This observation can be explained considering the higher hygroscopicity of GOS:MD matrices after equilibration at different RHs (Figure 1B). Furthermore, the addition of MD to commercial GOS allowed the obtaining of glassy matrices at 30°C and 44% RH (Figure 1A). In addition, samples storage led to an increase of the molecular mobility (Figure 2). This increased molecular mobility might be responsible for the collapse of GOS:MD, thus indicating that these conditions are not appropriate to preserve microorganisms viability during storage.

The *k* parameter obtained from the slopes of the lines in Figure 3 represents an important tool directly related with the product shelf-life at storage conditions (temperature and RH). This parameter has been previously determined for *Lactobacillus delbrueckii* subsp. *bulgarius* freeze-dried in GOS matrices (Tymczyszyn et al., 2012), *L. plantarum* and *L. delbrueckii* subsp *bulgaricus* immobilized in carboximethylcellullose films containing fructo-oligosaccharides (Romano et al., 2014), and for *Lactobacillus paracasei* freeze-dried in lactose matrices (Higl et al., 2007). The results obtained in this work clearly showed that storage at low temperatures and low RHs were the best conditions (lower *k* values) to stabilize *L. plantarum* CIDCA 83114 (Figure 3). These conditions warrants that at least 6–7 log CFU of viable microorganisms per gram of product are present after 90 days of storage (Figure 3A). This concentration was also warranted for microorganisms stored at 20°C at 11 and 22% RH up to 90 days, and stored at 30°C 11% RH up to 60 days (Figure 3). This supports their use in the formulation of products that are generally kept at room temperature (*i.e*., snacks, infant formulas, among others).

According to the European Food Safety Agency (EFSA), these values were reported as the minimum bacterial concentration that must be present in the product at the moment of being consumed (Aquilina et al., 2013; Phuapaiboon et al., 2013; Hill et al., 2014; Tripathi and Giri, 2014). In this context, *k* values represent an important tool to determine the shelf-life of spray-dried *L. plantarum* CIDCA 83114.

The correlation between *k* and T-T_g_ parameters (Figure 4) depicted a similar behavior as that observed previously for *Lactobacillus bulgaricus* freeze-dried in GOS matrices (Miao et al., 2008; Tymczyszyn et al., 2012). However, the increase of *k* observed in this work was dependent on the storage temperature (Figure 4). *L. paracasei* freeze-dried in lactose matrices depicted a similar behavior, the storage temperature *per se* having a strong influence on the stability of dehydrated samples (Higl et al., 2007).

When analyzed as a function of T-T_g_ parameter, the molecular mobility of the GOS:MD matrices (Figure 2) and *k* values of spray-dried *L. plantarum* CIDCA 83114 (Figure 4) showed different patterns. This indicates that the loss of viability at higher storage temperatures could not be explained only by the increase of molecular mobility, pointing out that other inactivation mechanisms are certainly present. In this regard, the reported higher penetration of oxygen in samples with higher oxidative effects appears as a plausible co-existent mechanism (Teixeira et al., 1996; Higl et al., 2007; Ying et al., 2011).

According to Figure 5, the effect of temperature on *k* is conditioned by the water activity. For example among samples equilibrated at 11% RH, the increase of *k* at different storage temperatures is lower than among samples with a higher water content (44% RH) (Figure 5). These results are consistent with those reported by Higl et al. (2007). They proposed that inactivation rates of low water activity samples are independent on the temperatures.

The linearization of the Arrhenius equation allowed determining the Ea for each storage condition from the slopes of the lines (Figure 5). In turn, the linear correlation between Ea and RH (Figure 6) allowed determining the stability of dehydrated samples exposed to different storage conditions during transport and commercialization. Although this equation has been widely used to predict the stability of dehydrated foods, it has been scarcely used to predict lactic acid bacteria stability at different storage conditions, and thus, it can be considered as an important contribution of this work (Ying et al., 2012; Muller et al., 2013).

## Conclusions

The obtained results strongly support the use of GOS:MD matrices as thermoprotectants of *L. plantarum* CIDCA 83114. Their thermophysical properties have been determined from an integrative perspective, thus supporting the determination of the most appropriate storage conditions of dehydrated samples. In spite of that, having appropriate thermophysical properties is a necessary but not sufficient condition for microbial stabilization. Bacterial recovery after drying processes is determined by the presence of protective molecules such as GOS, their stability being favored at high T-T_g_ values. At high RH or high storage temperatures (low T-T_g_ values), complex deteriorative reactions take place and lead to the increase of inactivation rates.

From a microbiological point of view, the results obtained support the production of *L. plantarum* CIDCA 83114, a potentially probiotic strain, at low cost, using GOS as thermoprotectants and spray-drying as dehydration method, with potential applications as functional food ingredient. Considering the inhibitory properties of the studied strain against *Escherichia coli* O157:H7, *Shigella* and *Salmonella* (Hugo et al., 2008; Golowczyc et al., 2011a; Kakisu et al., 2013a,b) and the high concentrations of GOS present in the matrices, the obtained microorganisms may be potentially considered as “synbiotic” products. Taking into account that prebiotics are not hydrolyzed in the upper part of the gastro-intestinal tract (definition of prebiotics, Gibson and Roberfroid, 1995), the consumption of potentially probiotic strains embedded in prebiotic matrices appears as an adequate strategy to protect microorganisms when exposed to the harmful conditions of the gastro-intestinal tract.

## Author contributions

NS and CS did the experimental work regarding water content determination, molecular mobility, and Tg determination. EG and MG did the experimental work and analyzed results obtained by spray-drying. ET and AGZ coordinated the work (analysis of results, discussion, and writing of the manuscript). All authors have approved the final version of the manuscript.

### Conflict of interest statement

The authors declare that the research was conducted in the absence of any commercial or financial relationships that could be construed as a potential conflict of interest.

## Footnotes

^1^Prebiotics are defined as “non-digestible food ingredients that beneficially affect the host by selectively stimulating the growth and/or activity of one or a limited number of bacteria in the colon, thus improving the host health” (Gibson and Roberfroid, 1995).

## Abbreviations

GOS: galacto-oligosaccharides
T_g_: glass transition temperature
MD: maltodextrin
DSC: differential scanning calorimetry
^1^H-NMR: proton nuclear magnetic resonance
RH: relative humidity
d.b.: dry basis
DP: degree of polymerization
FID: free induction decay analysis
CFU: cell forming units.
